# Serum Metabolic Profiling Reveals Potential Anti-Inflammatory Effects of the Intake of Black Ginseng Extracts in Beagle Dogs

**DOI:** 10.3390/molecules25163759

**Published:** 2020-08-18

**Authors:** Ye Jin Kim, Dae Young Lee, Ho-Eun Park, Dahye Yoon, Bumkyu Lee, Jae Geun Kim, Kyung-Hoan Im, Young-Seob Lee, Wan-Kyu Lee, Jae Kwang Kim

**Affiliations:** 1 Division of Life Sciences, College of Life Sciences and Bioengineering, Incheon National University, Yeonsugu, Incheon 22012, Korea; 201721047@inu.ac.kr (Y.J.K.); jgkim@inu.ac.kr (J.G.K.); khim61@inu.ac.kr (K.-H.I.); 2 Department of Herbal Crop Research, National Institute of Horticultural and Herbal Science, RDA, Eumseong 27709, Korea; dylee0809@gmail.com (D.Y.L.); dahyeyoon@korea.kr (D.Y.); youngseoblee@korea.kr (Y.-S.L.); 3 College of Veterinary Medicine, Chungbuk National University, Cheongju 28644, Korea; phu4793@naver.com (H.-E.P.); wklee@chungbuk.ac.kr (W.-K.L.); 4 Department of Environment Science & Biotechnology, Jeonju University, Jeonju 55069, Korea; leebk@jj.ac.kr

**Keywords:** black ginseng, serum, metabolic profiling, dog, metabolite

## Abstract

Black ginseng (BG) has better health benefits than white ginseng. The intake of BG changes the levels of metabolites, such as amino acids, fatty acids, and other metabolites. However, there is no research on the effect of BG extract intake on the metabolic profile of dog serum. In this study, serum metabolic profiling was conducted to investigate metabolic differences following the intake of BG extracts in beagle dogs. The beagle dogs were separated into three groups and fed either a regular diet (RD, control), RD with a medium concentration of BG extract (BG-M), or RD with a high concentration of BG extract (BG-H). Differences were observed among the three groups after the dogs ingested the experimental diet for eight weeks. The concentrations of alanine, leucine, isoleucine, and valine changed with the intake of BG extracts. Furthermore, levels of glycine and β-alanine increased in the BG-H group compared to the control and BG-M groups, indicating that BG extracts are associated with anti-inflammatory processes. Our study is the first to demonstrate the potential anti-inflammatory effect of BG extract in beagle dogs. Glycine and β-alanine are proposed as candidate serum biomarkers in dogs that can discriminate between the effects of ingesting BG-H.

## 1. Introduction

*Panax ginseng* C.A. Meyer is a famous traditional medicinal plant belonging to the Araliaceae family. Ginseng has been used for various therapeutic purposes in oriental traditional medicine and is now widely used in Asia. Recently, ginseng has been found to have a pharmacological effect on insulin regulation, obesity, cancer, inflammation, nervous system disorders, and so on [1,2,3,4,5]. Moreover, research has been conducted to develop methods to increase the pharmacological activity of ginseng by using thermal processing [6,7]. According to this concept, black ginseng (BG) is produced from white ginseng by heat treatment. BG requires five cycles of steaming (3 h for each cycle) at 98 °C, which makes it a unique black colour [8]. BG possesses better biological activity than white ginseng, inducing anti-stress, anti-cancer, anti-inflammation, and free radical scavenging pharmacological effects [9,10].

Inflammation is the root cause of many diseases [11]. Abnormal activation of acute inflammatory reactions can damage various tissues and organs, causing chronic inflammation. Continuous chronic inflammation can lead to life-threatening diseases such as bacterial sepsis, rheumatoid arthritis, skin inflammation, and cancer [12]. Various treatments have been developed to cure the inflammatory response by enhancing the immune system [13,14]. Among the treatments, since dietary and herbal therapies as complementary and alternative medicines contain natural substances that can safely promote health and relieve disease, research on dietary and herbal sources with anti-inflammatory effects has been actively performed [15,16,17]. Moreover, the anti-inflammatory response of black ginseng has already been demonstrated in many studies using inflammatory rat models [7,8,9,10]. Metabolomics studies are becoming more important to understand the mechanisms of anti-inflammation. A recent study has reported that serum metabolic profiling in a normal rat model after ginseng intake will help understand the metabolic mechanisms of the anti-inflammatory response to ginseng [18]. However, metabolomics studies on the effects of black ginseng in animals and humans are limited.

To understand the health effects of functional foods, comprehensive investigations have been performed using high-throughput analyses such as genomics, proteomics, and metabolomics. In particular, metabolomics, a new technique for quantitative and qualitative analysis of small metabolites (<1500 Da), is a combination of metabolic profiling and multivariate analysis. It is also suitable for distinguishing between phenotypes and finding metabolites that contribute to phenotypic differences [19,20]. In addition, metabolic profiling with mass spectrometry (MS) or nuclear magnetic resonance spectrometry (NMR) has been applied to evaluate the effect of ginseng on reducing obesity and inflammation [17,21,22]. It has been reported that the ingredients in ginseng, including ginsenosides, are poorly absorbed after oral administration in rats [23]. Beagle dogs are the most commonly used non-rodent species in preclinical evaluations and can be used to better characterise the effects of ginseng than rat models [23]. However, no studies have evaluated the metabolic changes on potential effectiveness of BG administration in dogs based on a metabolic approach.

Our aim was to assess the metabolic changes after the intake of BG extracts in beagle dogs. To accomplish this, serum metabolic profiling was performed using gas chromatography time-of-flight mass spectrometry (GC-TOF/MS) and GC-flame ionisation detection (GC-FID) with multivariate statistics. Furthermore, we aimed to identify serum metabolites as potential markers to determine the effects of BG extract intake in dogs.

## 2. Results and Discussion

### 2.1. Serum Metabolic Profiling

**Figure 1:**
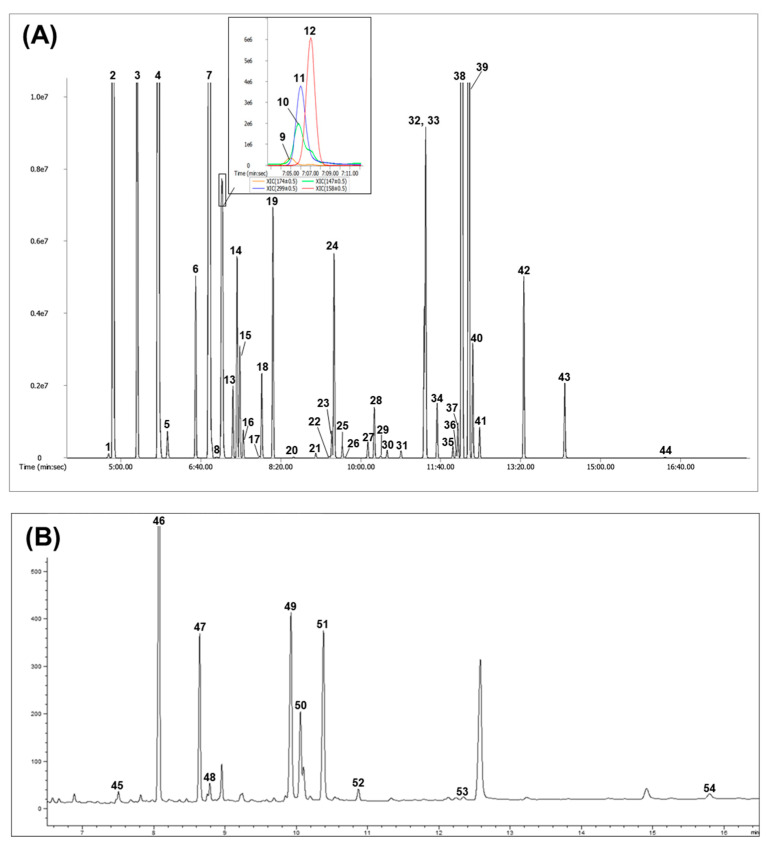
Representative gas chromatography time-of-flight mass spectrometry (GC-TOF MS) hydrophilic compounds (**A**), and GC-flame ionisation detection (GC-FID) fatty acids (**B**) chromatograms obtained from the serum of beagle dogs fed BG-H for 8 weeks. BG-H, a regular diet with high concentration of BG extract. Fatty acid formula is: C (number of carbon atoms): (number of double bonds) nx (position of double bonds). Peak: 1, pyruvic acid; 2, lactic acid; 3, alanine; 4, oxalic acid; 5, glycolic acid; 6, valine; 7, urea; 8, serine-1; 9, ethanolamine; 10, phosphoric acid; 11, glycerol; 12, leucine; 13, isoleucine; 14, proline; 15, glycine; 16, succinic acid; 17, fumaric acid; 18, serine-2; 19, threonine; 20, β-alanine; 21, malic acid; 22, aspartic acid; 23, methionine; 24, pyroglutamic acid; 25, threonic acid; 26, cysteine; 27, glutamic acid; 28, phenylalanine; 29, arabinose; 30, asparagine; 31, xylitol; 32, glutamine; 33, L-2-chlorophenylalanine (internal standard); 34, citric acid; 35, fructose-1; 36, fructose-2; 37, mannose; 38, glucose-1; 39, glucose-2; 40, lysine; 41, tyrosine; 42, inositol; 43, tryptophan; 44, sucrose; 45, C14:0 (myristic acid); 46, C15:0 (pentadecanoic acid, internal standard); 47, C16:0 (palmitic acid); 48, C16:1 (palmitoleic acid); 49, C18:0 (stearic acid); 50, C18:1 (oleic acid); 51, C18:2 (linoleic acid); 52, C18:3n3 (linolenic acid); 53, C20:3n6 (eicosatrienoic acid); 54, C22:5n3 (docosapentaenoic acid).

The biological activity of ginseng is associated with primary metabolites, such as amino acids, organic acids, and fatty acids [24]. However, how metabolites change in the serum of dogs after they are fed with BG extracts is poorly understood. Therefore, in our study, metabolic profiling was performed to evaluate the metabolic differences in the serum of beagle dogs fed with BG extracts. The GC-TOF/MS and GC-FID analyses identified a total of 49 metabolites, including nine long chain fatty acids (LCFAs), 21 amino acids, 10 organic acids, five sugars, one amine, and three sugar alcohols (Figure 1).

### 2.2. Multivariate Analyses

**Figure 2:**
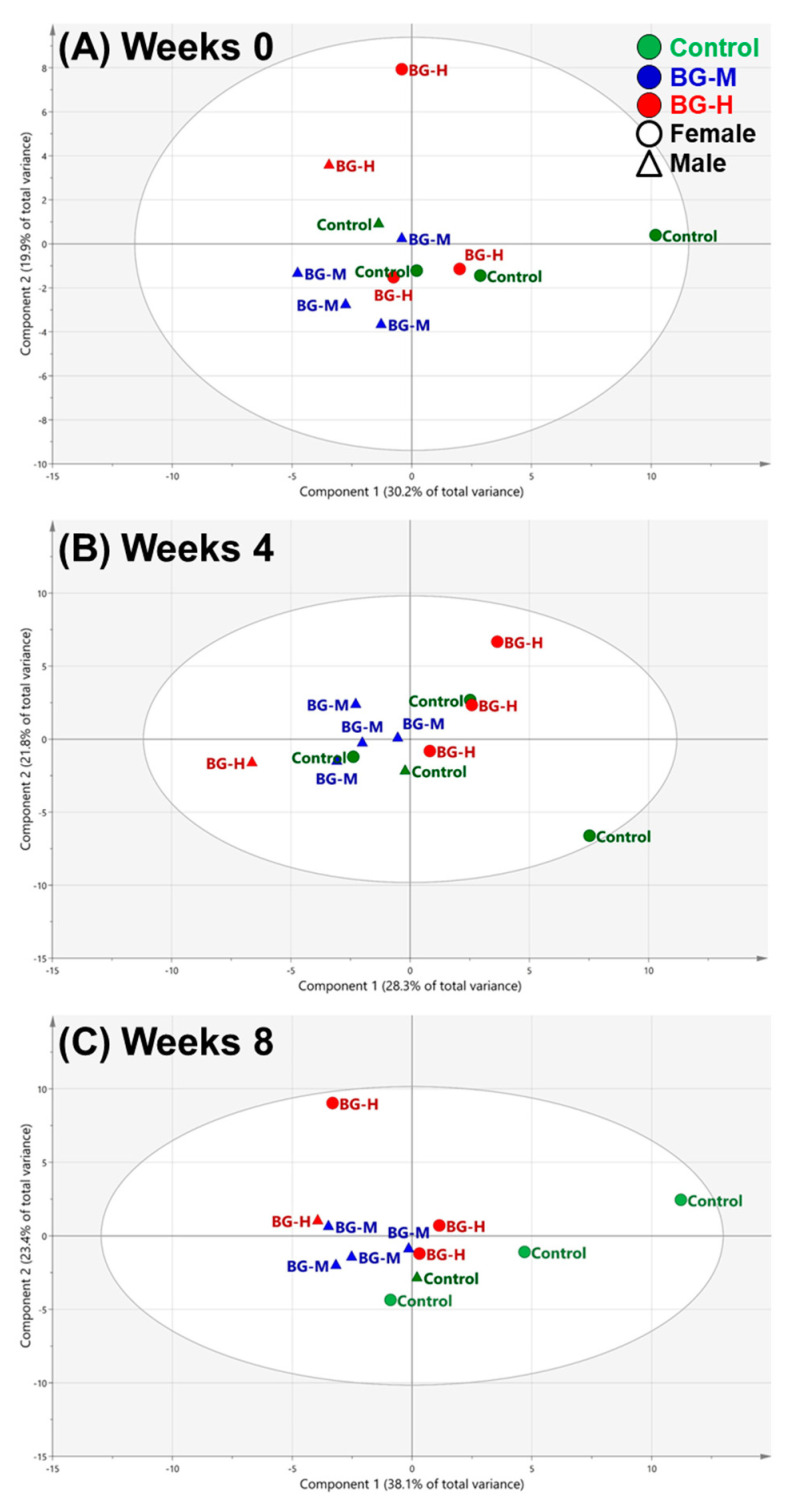
Score plots of the principal component analyses (PCA) models obtained from 49 metabolites in the serum of beagle dogs fed on a control, medium concentration of BG extract (BG-M), or BG-H diet at 0 (**A**), 4 (**B**), and 8 (**C**) weeks. Control, a regular diet; BG-M, regular diet with a medium concentration of BG extract; BG-H, regular diet with a high concentration of BG extract.

Multivariate analyses, such as principal component analyses (PCA) and orthogonal partial least squares discriminant analysis (OPLS-DA), were performed using the results of the serum metabolic profiling obtained from the control, BG medium dose (BG-M), and BG high dose (BG-H) groups. As a general unsupervised statistical analysis, PCA anticipates similarities and differences between numerous variables in the low-dimensional plane [25]. The original data is transformed into a new set of variables known as the principal component (PC) using an orthogonal linear transformation. In two-dimensional plots, the score and loading on the PC can identify the correlation between the patterns in the dataset and the variables. In the score plot of the PCA, each point represents an individual sample, and the distance between points is related to the similarity between samples. In the PCA score plot at week 0, no differences were observed between the three groups of dogs, suggesting that the physical state of each group was similar at the start of the experimental trials (Figure 2A). In addition, no metabolic differences were observed among the three groups after four weeks of the experiment (Figure 2B). However, after feeding the dogs for eight weeks, there were differences between the three groups (Figure 2C). In the score plot for week 8, two PCs of the score plot explained 61.5% of the total variance (component 1, 38.1%; component 2, 23.4%). Component 1 resolved the separation of the BG-H and BG-M groups from the control group. At the same time, no differences between two genders were observed in beagle dogs fed BG extracts for eight weeks (Figure 2). Overall, the PCA model was successfully applied to thoroughly investigate metabolic changes caused by the intake of BG extract for eight weeks. The results presented in the score plot for eight weeks indicate that the intake of moderate and high concentrations of BG extract alters the composition of metabolites in the serum of beagle dogs, with no significant difference between the sexes.

**Figure 3:**
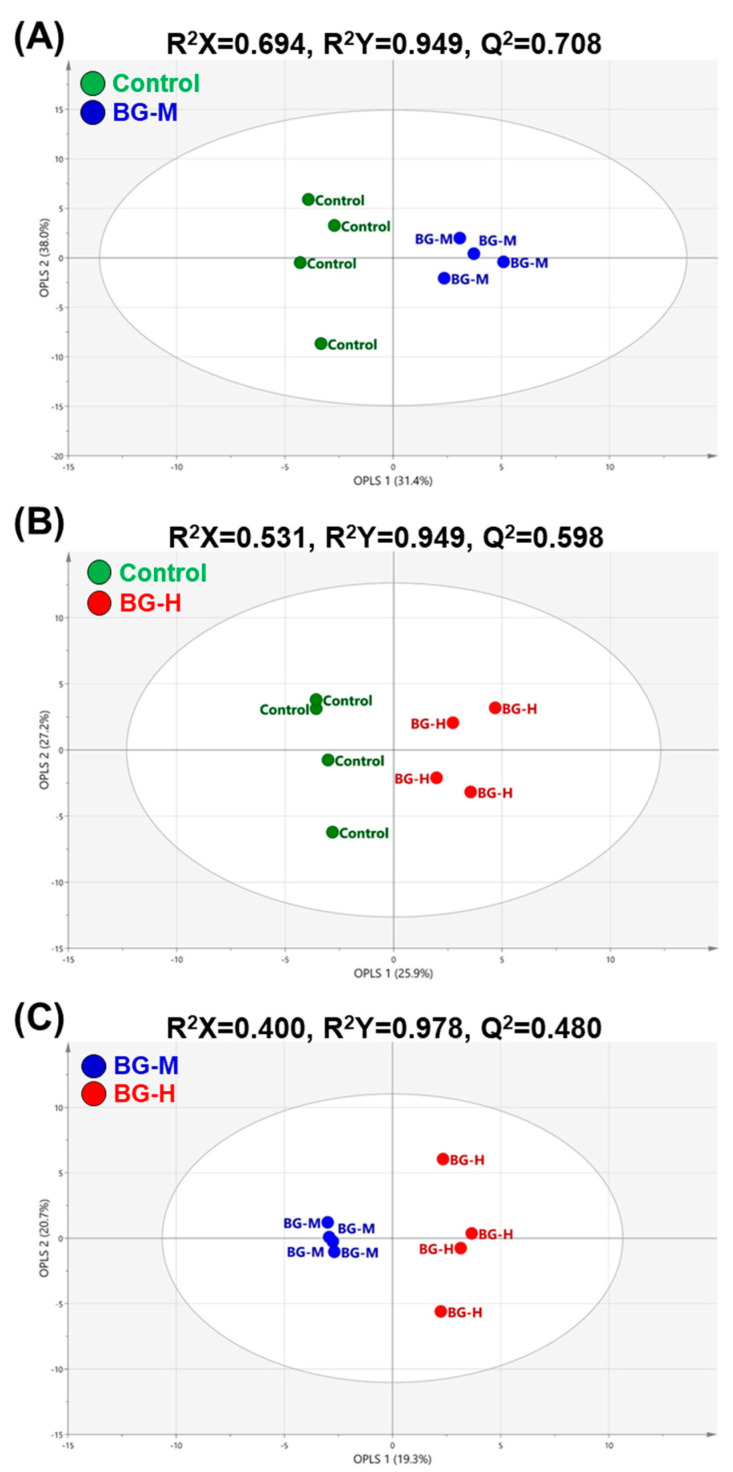
Score plots of the orthogonal partial least squares discriminant analysis (OPLS-DA) models obtained from 49 metabolites in the serum of beagle dogs fed on a control, BG-M, or BG-H diet for 8 weeks. OPLS-DA models indicate regressions between the control and BG-M (**A**), control and BG-H (**B**), or BG-M and BG-H (**C**) groups. Control, a regular diet; BG-M, regular diet with a medium concentration of BG extract; BG-H, regular diet with a high concentration of BG extract.

Supervised OPLS-DA is usually applied to distinguish between the data according to two known classes in the original dataset, and to find characteristic chemical markers [26]. The quality of the OPLS-DA model can be explained by the R^2^Y and Q^2^ values, which are the fit and predictability values of the model divided into two classes, respectively. Values of R^2^Y > 0.7 and Q^2^ > 0.5 are considered ‘good’. To investigate potential differences in serum metabolites between the BG-H or BG-M diets at week 8, multiple OPLS-DA models were acquired by comparing two groups: control and BG-M, control and BG-H, and BG-M and BG-H. Comparisons between the control and BG-M, and the control and BG-H groups were conducted to screen for metabolic changes in the serum related to the intake of BG extracts compared to the control group. The comparison between the BG-M and BG-H groups was performed to assess metabolic differences according to the intake of different concentrations of BG extract. The three-paired OPLS-DA models had R^2^Y values of 0.949–0.978 and Q^2^ values of 0.480–0.708. The control, BG-M, and BG-H groups were clustered separately in the score plot of each OPLS-DA model (Figure 3). These results indicate that serum metabolic profiling can reveal metabolic changes with the intake of BG extract at different concentrations.

**Figure 4:**
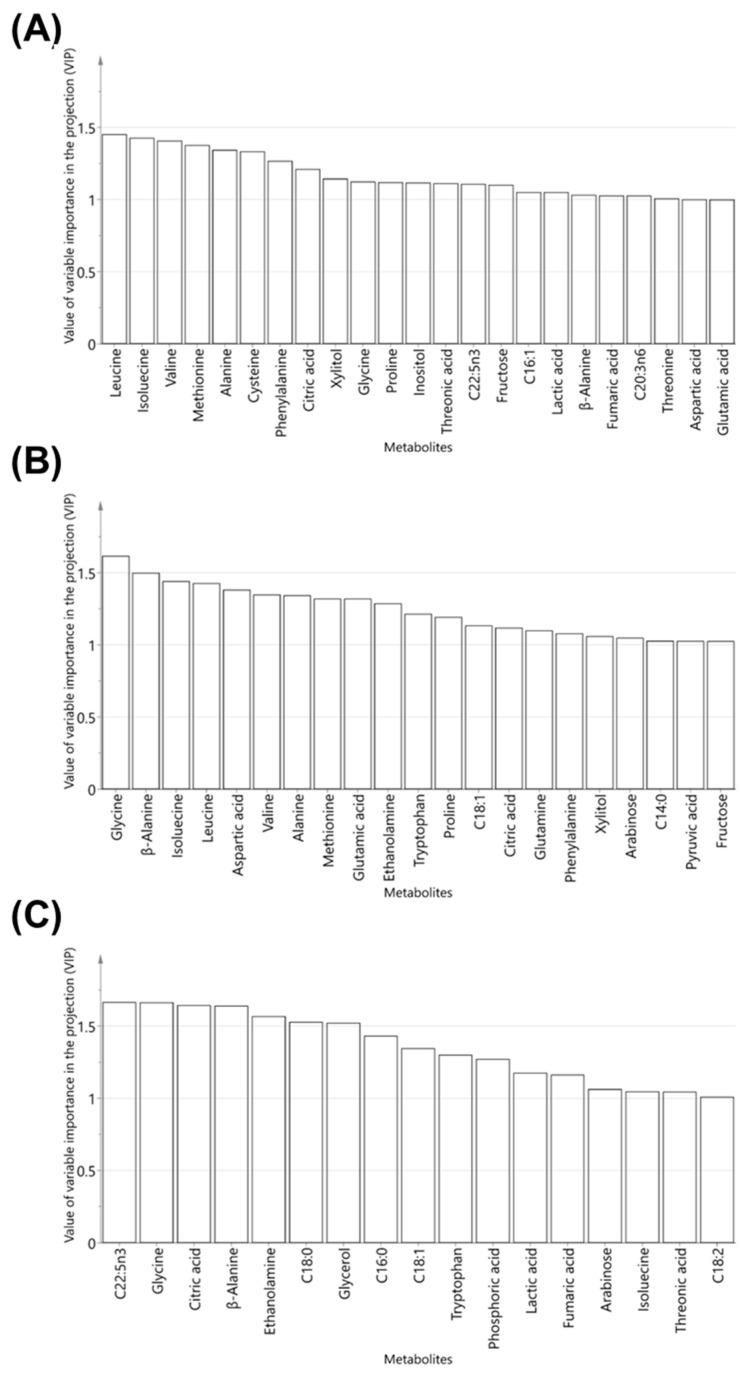
Variables important in the projection (VIP) plots of the orthogonal partial least squares discriminant analysis (OPLS-DA) models obtained from 49 metabolites in the serum of beagle dogs fed on a control, BG-M, or BG-H diet for 8 weeks. OPLS-DA models indicate regressions between the control and BG-M (**A**), control and BG-H (**B**), or BG-M and BG-H (**C**) groups. Metabolites with VIP values > 1.0 are presented in the VIP plots. Fatty acid formula is: C (number of carbon atoms): (number of double bonds) nx (position of double bonds). Control, a regular diet; BG-M, regular diet with a medium concentration of BG extract; BG-H, regular diet with a high concentration of BG extract; C14:0, myristic acid; C16:0, palmitic acid; C16:1, palmitoleic acid; C18:0, stearic acid; C18:1, oleic acid; C18:2, linoleic acid; C20:3n6, eicosatrienoic acid; C22:5n3, docosapentaenoic acid.

To investigate the serum metabolites that contribute significantly to the clustering pattern in three-paired OPLS-DA models, important variables in the projection (VIP) plots of the OPLS-DA model were acquired. In the VIP plot, the VIP value indicates the relative value of the contributing metabolites. The components with VIP values greater than 1.0 are considered important variables to the separation of groups [27]. In our study, 23 metabolites showed significant VIP values (>1.0) in the VIP plot of the control and BG-M groups. Among the metabolites, amino acids such as isoleucine, leucine, valine, methionine, and alanine had the highest VIP values (Figure 4A). In addition, 13 amino acids containing glycine, β-alanine, isoleucine, leucine, and valine were found to be important metabolites in the VIP plot of the control and BG-H groups (Figure 4B). When comparing the BG-M and BG-H groups in the OPLS-DA model, 17 serum metabolites containing C22:5n3, glycine, citric acid, β-alanine, and ethanolamine were identified as the highest contributors (Figure 4C). Consequently, BG extracts remarkably influenced the concentrations of amino acids, including glycine, β-alanine, isoleucine, leucine, and valine, in the serum of beagle dogs.

### 2.3. Identification of Metabolic Differences

**Figure 5:**
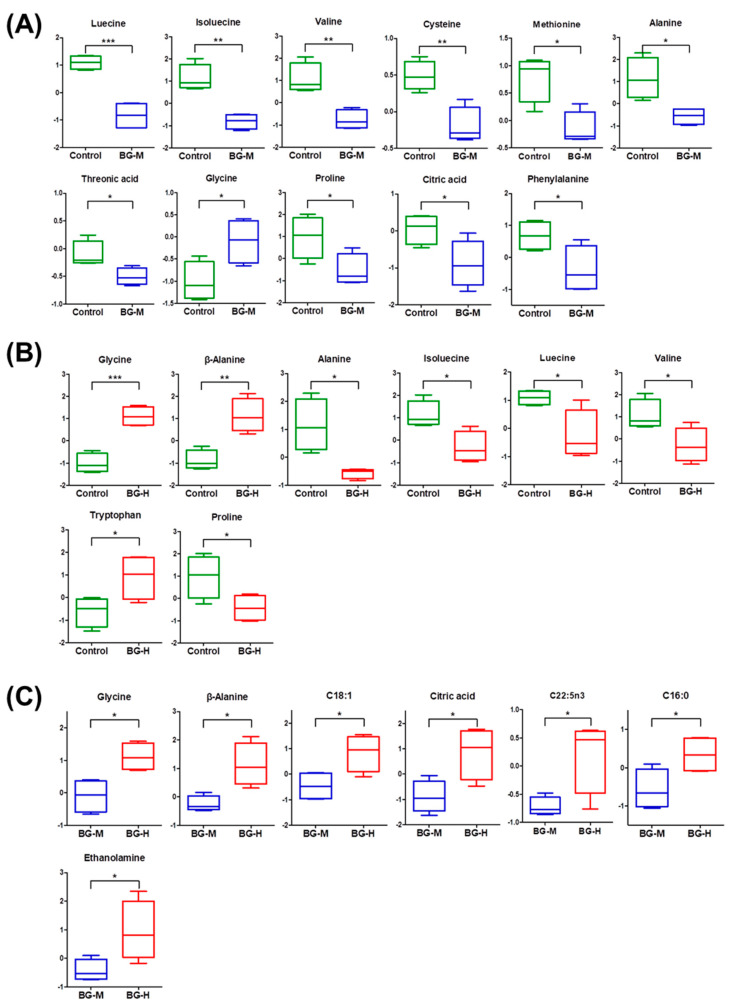
Box plots of metabolites that differed significantly (* *p* < 0.05, ** *p* < 0.01, *** *p* < 0.001) in the serum of beagle dogs between the control and BG-M groups (**A**), control and BG-H (**B**), and BG-M and BG-H groups (**C**). Fatty acid formula is: C (number of carbon atoms): (number of double bonds) nx (position of double bonds). Control, a regular diet; BG-M, regular diet with a medium concentration of BG extract; BG-H, regular diet with a high concentration of BG extract; C16:0, palmitic acid; C18:1, oleic acid; C22:5n3, docosapentaenoic acid.

To identify significant differences (*p* < 0.05) in the serum metabolites of beagle dogs after ingesting BG extracts for eight weeks, Student’s t-tests were conducted on metabolites with VIP values > 1.0. Significant differences (*p* < 0.05) in metabolites among the three diets were confirmed by box plots. In the comparison between the control and BG-M groups, 11 metabolites were identified as significantly different (Figure 5A). Of these metabolites, nine amino acids (isoleucine, leucine, valine, methionine, cysteine, threonic acid, proline, phenylalanine, and alanine) decreased and only glycine increased in the BG-M group compared to the control group (Figure 5A). In addition, in the comparison between the control and BG-H groups, there were significant differences in eight amino acids (Figure 5B). Higher levels of glycine and β-alanine were detected in the serum of beagle dogs fed with BG-H extract, while isoleucine, leucine, valine, alanine, and proline decreased in the BG-H group (Figure 5B). Amino acids are involved in tissue growth and maintenance and can be classified as alpha-(α-), beta-(β-), gamma-(γ-), or delta-(δ-) amino acids depending on the location of the core structural functional groups [28]. Twenty-one of the α-amino acids, including leucine, isoleucine, valine, and alanine, are involved in various biological activities. α-Amino acids play a role in the formation of proteins [29]. In particular, ginseng extract supplements are involved in the immune response by promoting the synthesis of serum proteins that require α-amino acids [30,31]. Based on these findings, it can be presumed that intake of moderate and high concentrations of BG extract increases the synthesis of serum proteins using α-amino acids, such as isoleucine, leucine, alanine, and valine. We propose that BG extract ingestion could potentially influence the anti-inflammatory process in beagle dogs.

In the comparison between the BG-M and BG-H groups, seven metabolites (glycine, β-alanine, C18:1, citric acid, C22:5n3, C16:0, and ethanolamine) were identified as significantly different (Figure 5C). In our results, the levels of glycine and β-alanine were the highest in the BG-H group compared to the other two groups. Glycine is not only one of the α-amino acids, but also an effective anti-inflammatory inducer, improving energy metabolism and immune function [32]. Moreover, β-alanine, a non-protein amino acid, is excluded from the α-amino acids. However, β-alanine has an immune function by promoting energy metabolism [33]. Thus, we suggest that a high concentration of BG extract shows a significant anti-inflammatory effect by inducing the production of glycine and β-alanine, and it improves the immune system in beagle dogs.

**Figure 6:**
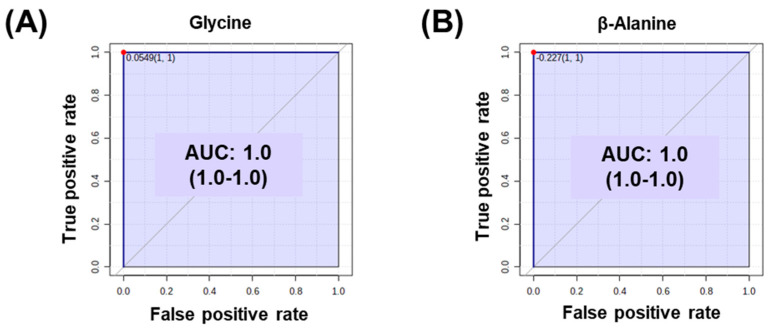
Receiver operating characteristic (ROC) curves of glycine (**A**) and β-alanine (**B**) that differed significantly in the serum of beagle dogs fed a regular diet with medium and high concentrations of black ginseng extract.

Furthermore, to verify glycine and β-alanine as potential serum biomarkers contributing to the anti-inflammatory effect caused by the ingestion of a high concentration of BG extract, receiver operating characteristic (ROC) analysis was performed on the BG-M and BG-H groups (Figure 6). Metabolomics is now widely recognised as a useful tool to identify biomarkers for the characterisation and diagnosis of many diseases [34]. Among the metabolomics methods, ROC curves show sensitivity, specificity, and area under the curve (AUC) value. AUC values indicate how well the two groups are separated. In general, AUC values of 0.9–1.0 are considered perfect, values of 0.7–0.9 are good, and values less than 0.5 have no differential power [34]. In our study, the AUC values of glycine and β-alanine were 1.0, indicating that they are good biomarkers for predicting a high concentration intake of BG extract. Therefore, we propose that glycine and β-alanine can be used as candidate serum biomarkers for identifying anti-inflammatory responses induced by the intake of high concentrations of BG extract. Consequently, the BG-M and BG-H groups showed changes in amino acids associated with potential anti-inflammatory processes when compared to the control group. In particular, the BG-H group had a better effect than the BG-M group.

### 2.4. Correlation between Serum Metabolites

**Figure 7:**
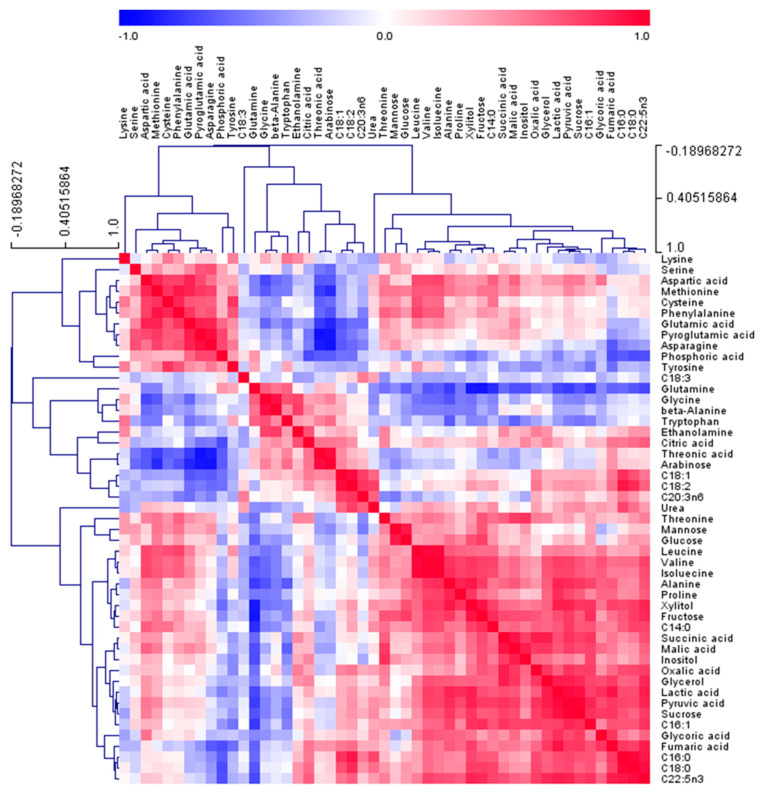
Correlation matrix of the 49 metabolites identified from the serum of beagle dogs. Each square represents the Pearson’s correlation coefficient by the intensity of the blue or red colour. Fatty acid formula is: C (number of carbon atoms): (number of double bonds) nx (position of double bonds). C14:0, myristic acid; C16:0, palmitic acid; C16:1, palmitoleic acid; C18:0, stearic acid; C18:1, oleic acid; C18:2, linoleic acid; C18:3, linolenic acid; C20:3n6, eicosatrienoic acid; C22:5n3, docosapentaenoic acid.

Pearson’s correlation analysis and hierarchical cluster analysis (HCA) were conducted to assess the correlations between metabolites among the 49 identified metabolites in the serum of beagle dogs (Figure 7). As the first amino acid group, leucine, isoleucine, valine, and alanine were positively clustered in the matrix (r = 0.6559–0.9902, *p* < 0.05). Glycine and β-alanine, as the second amino acid group, were also positively correlated (r = 0.9105, *p* < 0.0001). Moreover, the first and second amino acid groups were negatively correlated with each other (r = −0.6692–−0.4953, *p* < 0.1). These results support our opinion that BG-M and BG-H diets have a potential anti-inflammatory effect by inducing the use of leucine, isoleucine, valine, and alanine for the synthesis of serum proteins, and the BG-H diet promotes high levels of glycine and β-alanine production.

## 3. Materials and Methods

### 3.1. Plant Materials

BG products were processed using five-year-old *Panax ginseng* harvested from Eumseong-gun, Chungbuk Province, Korea. It was cultivated according to the protocol of the “Ginseng GAP Standard Cultivation Guide” developed by the Rural Development Administration (RDA), Republic of Korea. A voucher specimen (NIHHS1901) was deposited at the herbarium of the Department of Herbal Crop Research, National Institute of Horticultural and Herbal Science (NIHHS), RDA, Eumseong-gun, Republic of Korea.

### 3.2. Preparation of BG, Experimental Design, Serum Collection

Raw peeled ginseng was washed and dried in hot wind and sunlight. BG was produced by steaming three times at 95–98 °C for 3–5 h in a pottery apparatus, and then dried at 50 °C for 24 h (Figure S1). Fine BG was selected, dried, and powdered. Exactly 4 kg of powdered samples were refluxed two times with 32 L of water for 4 h in a water bath (Thermo Scientific™, Waltham, MA, USA) at 80 °C. The extracts were filtered through filter paper and concentrated by a vacuum evaporator (Eyela, Tokyo, Japan) until 10.8 brix. The ginsenosides of the BG extract were identified using high-performance liquid chromatography (HPLC). As shown in Figure S2, the ginsenosides Rg1, Rb1, Rg3, Rk1, and Rg5 were detected. BG extract contains high amounts of Rg5, which is known as the main ginsenoside of black ginseng [8]. Placebo tablets (control) were produced that did not contain BG extract but contained the same quantity of freeze-dried (LP30, Ilshin Biobase Co., Yangju, Korea) maltodextrin powder as the BG tablets (200 mg each), i.e., 185.27 mg of maltodextrin and 14.73 mg of BG powder.

In this study, 12 healthy beagle dogs (6 females and 6 males) were separated into 3 experimental groups of 4 individuals, namely a regular diet (RD) or control group, BG-M (400 mg/10 kg/day) intake group, and BG-H (800 mg/10 kg/day) intake group. The dogs were fed with the experimental diets once daily for 8 weeks. All animal experiments were approved by the Institutional Animal Care and Use Committee of Chungbuk National University (approval no. CBNUA-1218-18-01). One animal was kept per cage. Each animal was provided with 250 g per day of feed for laboratory dogs (Cargill Agri Purina Inc., Sungnam, Korea). The dogs were 2 to 3 years old, and the body weight range was 8–12 kg. Blood samples were collected from healthy beagle dogs 3 times on weeks 0, 4, and 8, respectively, using BD Vacutainer^®^ SST™ II Advance (BD Biosciences, San Jose, CA, USA). Serum was obtained by centrifugation at 3000 rpm for 15 min at 4 °C and stored at −20 °C until use.

### 3.3. Chemicals and Reagents

The chemical standards of l-2-chlorophenylalanine, pentadecanoic acid, and unsaturated fatty acid methyl ester mixes were purchased from Sigma-Aldrich (St. Louis, MO, USA) and Supelco (Bellefonte, PA, USA). The silylating reagents of methoxylamine hydrochloride (MOX) and *N*-*O*-bis-(trimethylsilyl)-trifluoroacetamide + trimethylchlorosilane (BSTFA + 1% TMCS) were purchased from Sigma-Aldrich (St. Louis, MO, USA) and Tokyo Chemical Industry (TCI) Chemicals (Kita-ku, Tokyo, Japan), respectively. Chromatographic pure hexane, methanol, chloroform, and deionized water (DW) were procured from J.T. Baker, Inc. (Phillipsburg, NJ, USA). Toluene (≥99.8%, Sigma-Aldrich, St. Louis, MO, USA) and pentane (≥99%, TCI Chemicals, Kita-ku, Tokyo, Japan) were used as received.

### 3.4. Analysis of Hydrophilic Compounds

Hydrophilic compounds in the serum of beagle dogs were extracted and analysed by modifying previously described methods [35]. The serum (0.05 mL) was extracted with 0.15 mL of methanol:chloroform solution (3:1, *v*/*v*) and 0.03 mL of l-2-chlorophenylalanine as the internal standard (IS, 0.3 mg/mL). The sample was sonicated for 10 min, and then centrifuged at 14,000× *g*, for 15 min at 4 °C. The supernatant was transferred to a clean tube and concentrated with a speed-vac concentrator (VS-802F, Visionbionex, Gyeonggi, Korea) for ~3 h. To derivatise the sample, 0.08 mL of MOX (20 mg/mL) was added and reacted at 1200 rpm for 90 min at 37 °C. The sample was mixed with 0.08 mL of BSTFA + 1% TMCS and heated again at 60 °C and 1200 rpm for 60 min. Finally, the derivatised sample was transferred to a 2 mL autosampler vial and injected directly for GC-TOF/MS analysis. Agilent 7890B GC system (Agilent, Santa Clara, CA, USA) equipped with a CP-Sil 8 CB Low Bleed/MS column (30 m × 0.25 mm × 0.25 μm; CP 5860, Agilent, Santa Clara, CA, USA) and LECO Pegasus BT TOF mass spectrometer (LECO, St. Joseph, MI, USA) separated the hydrophilic metabolites in the serum. The injected volume was 1 μL, and split ratio was 1:25. The column temperature programme was started at 80 °C and held for 2 min, then increased to 320 °C a rate of 15 °C/min and maintained for 10 min. Front inlet and transfer line temperatures were set at 230 and 280 °C, respectively. A flow rate of carrier gas (helium) was 1 mL/min, and scanned mass range was 85–600 *m*/*z*. Peak identification of the GC-TOF/MS data were performed by comparing their retention times and mass spectrum with standard compounds, in-house library, and MS library (Nist and Wiley 9). Quantitative analysis was performed using the ratio of the analyte peak area to IS peak area.

### 3.5. Analysis of LCFAs

A previously described method was used to analyse LCFA in the serum [36]. Here, 2.5 mL of chloroform:methanol (2:1, *v*/*v*) solution and 0.1 mL of pentadecanoic acid (IS, 1 mg/mL) were added to the serum (0.1 mL) in a 15 mL tube. The sample was sonicated for 20 min. After sonication, 2.5 mL of 0.58% sodium chloride in DW was added into the tube. The mixture was vortexed for 20 s and then centrifuged at 15,000× *g* and 4 °C for 5 min. The lower layer (chloroform phase) of the tube was separated to a new 2 mL tube and dried using a speed-vac concentrator. To methylate the sample, the concentrated sample was mixed with 0.02 mL of 5 M sodium hydroxide, 0.18 mL of methanol, and 0.1 mL of toluene at 300 rpm and 85 °C for 5 min. Subsequently, 0.3 mL of boron trifluoride was added to the tube and reacted again under the same conditions. After methylation, 0.4 mL of DW and 0.8 mL of pentane were added and blended with the sample for 20 s. The mixture was centrifuged at 350× *g* and 4 °C for 15 min. The upper layer in the tubes was transferred into a clean 2 mL tube and then dried using a speed-vac concentrator. The dried sample was dissolved with 0.1 mL of hexane. The sample was filtered through a 0.5 μm syringe filter, and analysed by GC-FID. Agilent 7890A gas chromatograph equipped with a DB-Wax column (30 m × 0.25 mm × 0.25 μm; 122-7032, Agilent, Santa Clara, CA, USA) and a 7890 GC detector separated the fatty acids in the serum. The flow rate of carrier gas (nitrogen) was 1 mL/min. Front inlet and detector temperatures were set at 250 °C. The column temperature conditions were as follows: initial temperature was maintained at 130 °C for 3 min, raised to 230 °C at a rate of 20 °C/min, and the final temperature was increased to 250 °C at a rate of 3 °C/min and maintained for 5 min. Qualitative and quantitative analyses of LCFAs were conducted using saturated and unsaturated fatty acid methyl ester mixes.

### 3.6. Statistical Analysis

All quantified data were scaled with unit variance scaling prior to analysis. To evaluate metabolic changes in the serum from dogs fed three different diets, PCA and OPLS-DA were conducted using SIMCA-P version 14.1 (Umetrics, Umeå, Sweden). To identify significant differences (*p* < 0.05) after the intake of BG extracts for 8 weeks, Student’s t-tests were performed. ROC analysis was conducted by MetaboAnalyst 4.0 (www.metaboanalyst.ca). Box plots were constructed in GraphPad Prism 5 software (San Diego, CA, USA). Pearson’s correlation analyses were performed using SAS software 9.4 (SAS Institute Inc., Cary, NC, USA). Multi-Experiment Viewer version 4.9.0 (MeV) was used to construct HCA.

## 4. Conclusions

In summary, we used serum metabolic profiling to analyse 49 metabolites in beagle dogs fed with BG extracts. Metabolic differences were confirmed by multivariate analyses such as PCA and OPLS-DA. In the BG-H and BG-M groups, the concentrations of isoleucine, leucine, alanine, and valine were detected at lower levels than those in the control group. Glycine and β-alanine were higher in the BG-H group than that in the BG-M and control groups. To our knowledge, the present study is the first to suggest that the intake of BG extracts has a potential anti-inflammatory effect in beagle dogs based on serum metabolic profiling. Notably, we found changes in amino acid composition in the serum of beagle dogs fed a diet supplemented with BG-H and BG-M. In addition, we propose that glycine and β-alanine can be used as candidate serum biomarkers, implying that the consumption of a BG-H diet improves the immune system more than a BG-M diet. The results of our study together with the metabolic approach used may be a useful tool for understanding metabolism affected by the ingestion of BG extracts. An interesting aspect for future study is that black ginseng consumption will affect the composition of more diverse metabolites in animal models of inflammatory disease compared to normal models. Therefore, we intend to investigate dynamic changes of metabolites using metabolic profiling in animal models of inflammatory disease fed with a diet containing black ginseng.

## Supplementary Materials

The following are available online. Figure S1: Black ginseng that has been dried three times after being steam cooked three times, Figure S2: Identification of ginsenosides in black ginseng extract by high-performance liquid chromatography (HPLC) analysis.
