# Supplementary material for: Serum Metabolic Profiling Reveals Potential Anti-Inflammatory Effects of the Intake of Black Ginseng Extracts in Beagle Dogs
Source: Molecules. 2020 Aug 18;25(16):3759. doi: 10.3390/molecules25163759 (PMC7465512; doi:10.3390/molecules25163759)
Supplement: Supplementary file 1 [file molecules-25-03759-s001.pdf]

Serum Metabolic Profiling Reveals Potential Anti-Inflammatory Effects of the Intake of Black Ginseng Extracts in Beagle Dogs

Ye Jin Kim [1,†], Dae Young Lee [2,†], Ho-Eun Park [3], Dahye Yoon [2], Bumkyu Lee [4], Jae Geun Kim [1], Kyung-Hoan Im [1], Young-Seob Lee [2], Wan-Kyu Lee[3], and Jae Kwang Kim [1,*]

[1] Division of Life Sciences, College of Life Sciences and Bioengineering, Incheon National University, Yeonsugu, Incheon 22012, Republic of Korea; 201721047@inu.ac.kr (Y.J.K.); jgkim@inu.ac.kr (J.G.K.); khim61@inu.ac.kr (K.-H.I.); kjkpj@inu.ac.kr (J.K.K.)

[2] Department of Herbal Crop Research, National Institute of Horticultural and Herbal Science, RDA, Eumseong 27709, Republic of Korea; dylee0809@gmail.com (D.Y.L.); dahyeyoon@korea.kr (D.Y.); youngseoblee@korea.kr (Y.-S.L.)

[3] College of Veterinary Medicine, Chungbuk National University, Cheongju 28644, Republic of Korea; phu4793@naver.com (H.-E.P.); wklee@chungbuk.ac.kr (W.-K.L.)

[4] Department of Environment Science & Biotechnology, Jeonju University, Jeonju 55069, Republic of Korea; leebk@jj.ac.kr (B.L.)

* Correspondence: kjkpj@inu.ac.kr; Tel: +82-32-835-8241

† These authors contributed equally to this work.

Supplementary File

Figure S1. Black ginseng that has been dried three times after being steam cooked three times

Figure S2. Identification of ginsenosides in black ginseng extract by high-performance liquid chromatography (HPLC) analysis.

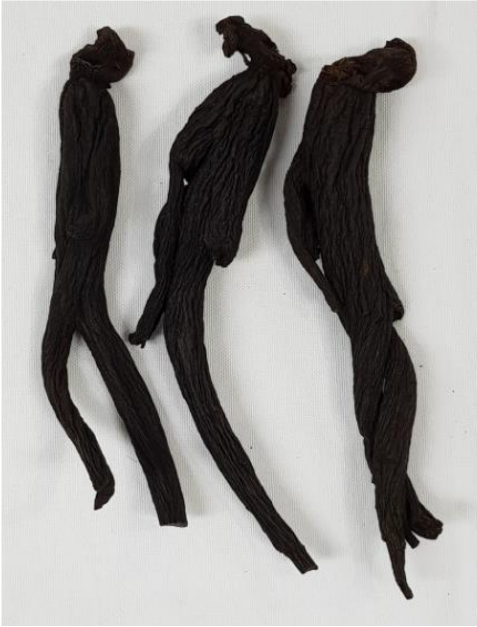

Figure S1. Black ginseng that has been dried three times after being steam cooked three times.

(A) Standard mixture

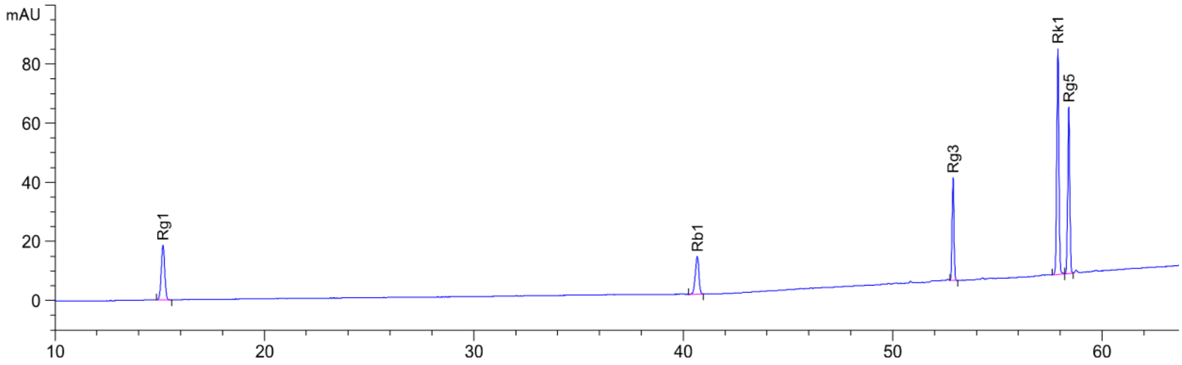

(B) Extract of black ginseng

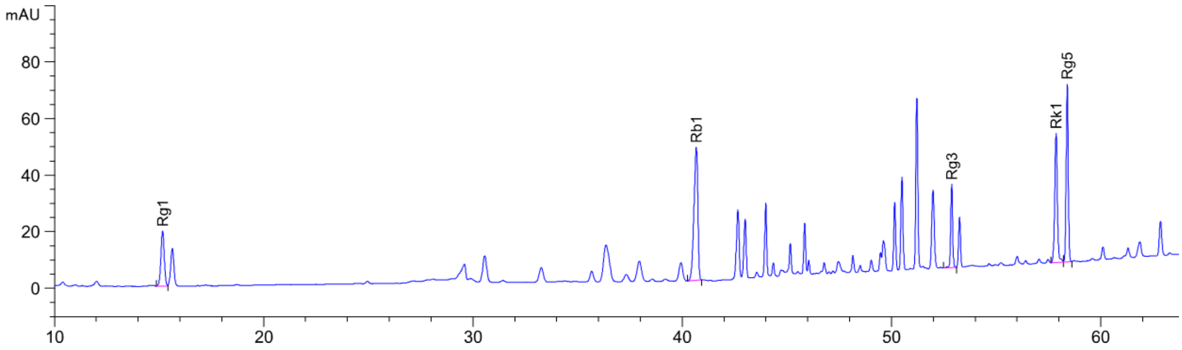

Figure S2. Identification of ginsenosides in black ginseng extract by high-performance liquid chromatography (HPLC) analysis.
